# Abnormal Functional Hierarchies of EEG Networks in Familial and Sporadic Prodromal Alzheimer's Disease During Visual Short-Term Memory Binding

**DOI:** 10.3389/fnimg.2022.883968

**Published:** 2022-06-17

**Authors:** Keith M. Smith, John M. Starr, Javier Escudero, Agustin Ibañez, Mario A. Parra

**Affiliations:** ^1^Department of Physics and Mathematics, School of Science and Technology, Nottingham Trent University, Nottingham, United Kingdom; ^2^Alzheimer Scotland Dementia Research Centre, School of Philosophy, Psychology and Language Sciences, University of Edinburgh, Edinburgh, United Kingdom; ^3^School of Engineering, Institute for Digital Communications, University of Edinburgh, Edinburgh, United Kingdom; ^4^Latin American Brain Health Institute (BrainLat), Universidad Adolfo Ibañez, Santiago, Chile; ^5^National Scientific and Technical Research Council (CONICET), Buenos Aires, Argentina; ^6^Center for Social and Cognitive Neuroscience, School of Psychology, Universidad Adolfo Ibáñez, Santiago, Chile; ^7^Global Brain Health Institute, UCSF, San Francisco, CA, United States; ^8^Trinity College Institute of Neuroscience, Trintity College Dublin, Dublin, Ireland; ^9^School of Psychological Sciences and Health, University of Strathclyde, Glasgow, United Kingdom; ^10^Neuroprogressive and Dementia Network, NHS Scotland, Glasgow, United Kingdom

**Keywords:** Alzheimer's disease, brain networks, aging, working memory, electroencephalogram (EEG)

## Abstract

Alzheimer's Disease (AD) shows both complex alterations of functional dependencies between brain regions and a decreased ability to perform Visual Short-Term Memory Binding (VSTMB) tasks. Recent advances in network neuroscience toward understanding the complexity of hierarchical brain function here enables us to establish a link between these two phenomena. Here, we study data on two types of dementia at Mild Cognitive Impairment (MCI) stage—familial AD patients (E280A mutation of the presenilin-1 gene) and elderly MCI patients at high risk of sporadic AD, both with age-matched controls. We analyzed Electroencephalogram (EEG) signals recorded during the performance of Visual Short-Term Memory (VSTM) tasks by these participants. Functional connectivity was computed using the phase-lag index in Alpha and Beta; and network analysis was employed using network indices of hierarchical spread (degree variance) and complexity. Hierarchical characteristics of EEG functional connectivity networks revealed abnormal patterns in familial MCI VSTMB function and sporadic MCI VSTMB function. The middle-aged familial MCI binding network displayed a larger degree variance in lower Beta compared to healthy controls (*p* = *0.0051*, Cohen's *d* = 1.0124), while the elderly sporadic MCI binding network displayed greater hierarchical complexity in Alpha (*p* = *0.0140*, Cohen's *d* = 1.1627). Characteristics in healthy aging were not shown to differ. These results indicate that activity in MCI exhibits cross-frequency network reorganization characterized by increased heterogeneity of node roles in the functional hierarchy. Aging itself is not found to cause VSTM functional hierarchy differences.

## Introduction

Functional disconnections caused by Alzheimer's disease (AD) can be characterized using brain network methodologies of patients and those at risk of this type of dementia (Badhwar et al., 2017). Yet, key issues remain unsolved including their validity for assessment of both familial AD (commonly used to model pre-clinical phases of the disease) and far more prevalent sporadic AD, and their ability to separate changes due to aging from those caused by the neurodegeneration (Ibáñez and Parra, 2014). The current study focuses on these outstanding issues through the implementation of a novel EEG functional connectivity methodology that unveils network topology changes (i.e., hierarchical spread and hierarchical complexity; Smith and Escudero, 2017) during performance of a memory task considered a marker for AD—the Visual Short-Term Memory Binding Test (VSTMBT) (Costa et al., 2017).

Although the links between genotype and phenotype in AD remain to be elucidated, earlier reports suggested that the E280A-PSEN1 mutation variant, presents clinically similarly to the sporadic late-onset AD (FAD) (Lopera et al., 1997; Acosta-Baena et al., 2011). Indeed, previous studies have consistently demonstrated that the E280A-PSEN1 FAD and sporadic AD (SAD) share a memory binding phenotype (Parra et al., 2010a,b). In fact, using EEG to analyse the ERP linked to this memory function in cases at risk of SAD and E280A-PSEN1 FAD, it has been demonstrated that these risk variants are indistinguishable both behaviorally and electrophysiologically (Pietto et al., 2016). We have highlighted the value of such evidence as it indicates that short-term memory binding impairments and their neural correlates are AD features shared across sporadic and genetic (i.e., E280A-PSEN1) variants.

Small-world deviations in AD from healthy aging in EEG connectivity have been related to a loss of complexity and efficiency (Stam et al., 2007a, 2009; De Haan et al., 2009). Progressively, investigations have looked into the key role of the deterioration of network hubs related to degradation of functional integration due to pathology (Buckner et al., 2009; Stam et al., 2009; Miao et al., 2011; De Haan et al., 2012), with more disruptions of complex functional network degree hierarchies, such as loss of assortativity (De Haan et al., 2009) and loss of hub connectivity to distant nodes (Liu et al., 2014; Dai et al., 2015), being found. Given this, we look specifically into the hierarchical layout of functional networks of neurodegeneration in terms of degree variance (hierarchical spread) and hierarchical complexity (Smith and Escudero, 2017).

Although functional connectivity of memory tasks in AD has been documented (Pijnenburg et al., 2004; Sperling et al., 2010), little is known of the effect of AD on the topology of working memory networks underpinning the impaired function of VSTMB and of age-related factors which separate familial and sporadic forms of the disease. Studying VSTMB in familial and sporadic Mild Cognitive Impairment (MCI) can unveil previously unknown features of the disconnecting pathology caused by AD which could expand recent findings from brain connectivity studies (Parra et al., 2017; Smith et al., 2017b) and shed new light on AD's key clinical manifestation of memory decline together with other cognitive impairments.

Moreover, the extent to which impairments found in specific cognitive functions in patients with AD can be solely attributed to the disease process and not to the normal course of aging remains little understood (Bondi et al., 2003; Spaan et al., 2003; Wakefield et al., 2014; Spaan, 2016). Evidence has accrued indicating that aging spares some cognitive systems while affecting others (Grady, 2008; Logie and Maylor, 2009; Reuter-Lorenz and Park, 2014). One such age-insensitive system is that subserving VSTMB (Brockmole et al., 2008; Parra et al., 2009; Read et al., 2016; Hoefeijzers et al., 2017; Rhodes et al., 2017).

We hypothesize that: (1) Those embarked on the course of familial and sporadic variants of AD will exhibit notable deviations in their VSTMB functions, (2) that such deviations will be accounted for by changes in network topology which would be similar across variants of risk of AD, and (3) VSTMB functional dependencies will not exhibit age-related changes.

## Materials and Methods

### Participants

The data in this study have been used in two previous studies (Pietto et al., 2016; Parra et al., 2017). It consists of people with familial MCI and their controls and people with sporadic MCI and their controls, detailed separately below. In both cases patients were evaluated with the Mini-Mental State Examination (MMSE) with results previously described (Pietto et al., 2016). The tasks were performed in an electrically shielded room with dim lighting. Participants sat comfortably at a desk facing the task display screen. The subjects were checked to ensure that none had a history of psychiatric or neurological diseases.

The familial MCI data consisted of 10 patients diagnosed with MCI (age 44.4 ± 3.2, years of education 7.3 ± 4.1) and 10 healthy controls (age 44.3 ± 5.6, years of education 6.8 ± 2.9) from Antioquia, Colombia. Each patient carried the mutation E280A of the presenilin-1 gene which leads to familial AD in 100% of carriers. The data consist of sixty-channel EEG activity recorded with a 64 channel EEG cap using SynAmps 2.5 in Neuroscan at 500 Hz and bandpass filtered from 1 to 100 Hz with impedances below 10 KΩ. Four ocular channels were discarded after being used to factor out oculomotor artifacts. The patients had not yet developed clinical symptoms warranting a diagnosis of dementia. Analysis of power-frequency spectrum showed that this data had been subject to a low pass filter with cut-off at 20 Hz.

### EEG Recordings

The sporadic MCI data consisted of 13 patients diagnosed with MCI (age 73.1 ± 9.0, years of education 14.1 ± 4.4) and 19 healthy controls (age 67.2 ± 10.14, years of education 16.5 ± 2.0) recruited from the Institute of Cognitive Neurology (INECO), Buenos Aires, Argentina. Criteria implemented for diagnosis derived from Petersen (2004) and Winblad et al. (2004). Nine of the patients were at particularly high risk from AD conversion having been classified as single or multi-domain amnestic MCI while three classified as non-amnestic MCI multi-domain (Mitchell et al., 2009). The data consist of EEG activity recorded with a Biosemi Active 128-channel Two system at 512 Hz and bandpass filtered from 1 to 100 Hz. This was then downsampled to 256 Hz.

For both datasets we use data from the encoding period during the performance of shape only and shape-color binding tests since deficits at this stage seem to be responsible for the VSTM binding problems found in AD (Parra et al., 2017). This consists of 1.2 s of continuous activity with 0.2 s pre-stimulus. Signals were re-referenced to an average reference before proceeding, following (Chella et al., 2016). Further oculomotor artifacts were removed using visual inspection and independent component analysis and epochs with other artifacts exceeding ±100 μV were discarded as detailed in Pietto et al. (2016). We seek to uncover underlying physiological substrates of the impaired binding function. In this way incorrect responses are not informative so only the trials where the subject responded correctly are included (Pietto et al., 2016).

### Behavioral Traits

From a neuropsychological perspective, the two groups of patients presented with similar backgrounds, Table 1. Both groups showed similar level of global cognitive impairment as denoted by the MMSE with instrumental abilities (IADL) denoting very mild but similar level of impairment. Memory and executive functions were affected in both groups as denoted by the recall of the Rey Figure and Fluency Tests. Attention was preserved in both groups as informed by the TMT-A (see Pietto et al., 2016 for details on performance). Taken together these data suggest that both groups were in very similar stages of multiple-domain amnesic MCI (maMCI) (Albert et al., 2011).

**Table 1:** Results of neuropsychiatric tests presented to both groups for patients and controls (mean ± standard deviation).

**Test**	**Familial MCI**	**Sporadic MCI**	**Familial control**	**Sporadic control**
MMSE	25.20 ± 4.50	26.46 ± 2.47	29.10 ± 1.10	29.50 ±0.52
IADL	7.2 ± 1.00	6.38 ± 1.06		
Rey figure-copy	21.89 ± 5.03	30.42 ± 4.58	26.38 ± 4.99	32.16 ± 5.80
Rey figure-recall	7.33 ± 4.89	11.04 ± 6.36	14.32 ± 5.18	16.49 ± 6.55
TMT-A	87.75 ± 38.30	59.23 ± 24.37	73.67 ± 26.44	42.63 ± 25.87

*MMSE, Mini-mental state examination; IADL, Instrumental activities of daily living scale; TMT-A, Trail-making test (part A)*.

### Ethics Committee Approval

All participants provided written informed consent in agreement with the Helsinki declaration and the studies were approved by the Ethics Committees of the University of Antioquia and INECO.

### Visual Short-Term Memory Tasks

**Figure 1:**
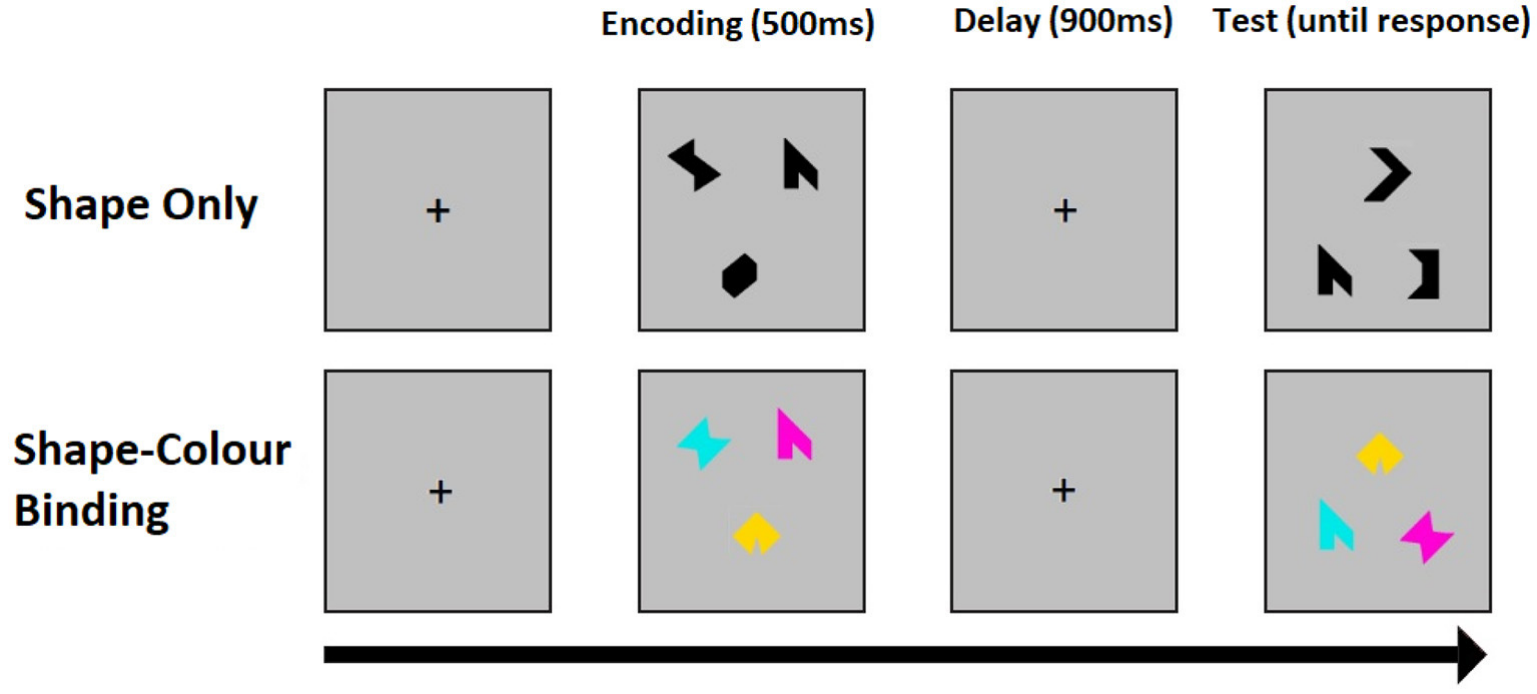
An example trial for the shape only **(top)** and shape-color binding **(bottom)** condition of the VSTM binding test. The test was synchronized with EEG recordings.

The binding function of visual short-term memory (VSTM) is singled out by contrasting tasks for the recognition of colored shapes, which requires binding of shape and color in memory retention (binding), and the recognition of single shapes which only requires the retention of constituent features. In the change detection task assessment of VSTM for shape alone, the arrays consist of three different black shapes and in the binding task the arrays consist of three different shapes each with a different color. Each task trial consists of an encoding period (500 ms), during which a study array is displayed on screen, followed by an unfilled short delay (900 ms) and test period with a test array. During the test period, participants are prompted to respond whether or not the objects in the two arrays are identical. The positions of the objects are randomized between arrays to avoid use of location as a memory cue. Both shapes and colors are chosen randomly for each trial from a set of eight shapes and a set of eight colors. A randomly chosen fifty percent of the trials have the same objects in both arrays. In the other 50 percent, two shapes seen during the encoding periods are replaced with two new shapes selected from the set, whereas in the binding task two colored shapes of the test display swap the colors they had during the encoding period. All participants start with a brief practice session before undergoing one hundred trials per task. Binding and shape tasks are delivered in a counterbalanced order across participants. Figure 1 shows an example trial for the two conditions of the VSTM binding task.

Both groups showed similar level of performance across task conditions i.e., Shape Only vs. Shape-Color Binding (MCI *Z* = 1.54 and their Controls *Z* = 1.42; MCI-FAD *Z* = 1.63 and their controls *Z* = 1.17; see Pietto et al., 2016). While MCI patients showed poorer performance than controls on both conditions of the STM binding test, MCI-FAD patients showed poorer performance than controls only on the Shape-Color binding condition. The source of such a discrepancy has been recently addressed (Parra et al., 2019). Assessing older samples (i.e., MCI) with large set sizes (3 items) might reduce the discrepancy classically reported between the two conditions of the STM binding test. The authors argued that this does not undermine the specificity of the Shape-Color binding condition for AD but reflects the influence that memory load exerts on patients with more advanced cognitive impairments (i.e., maMCI) (Petersen, 2004)—see also Parra et al. (2010a,b). This is reinforced by the observation that the two groups presented with a very similar profile of Shape-Color Binding impairment (Mann–Whitney U: 63, *Z* = −0.09, *p* = 0.93, *d* = 0.02; see Pietto et al., 2016).

### Functional Connectivity Networks

The Phase-Lag Index (PLI) was computed to assess the phase-dependent functional connectivity of the EEG channels (Stam et al., 2007b). This measures the strength and consistency of pairwise lead/lag relationships of electrode activity of the brain's electromagnetic pulses. Such phase-based measures are particularly useful for assessing interregional dependencies from EEG due to their immunity to the volume conduction effect. Alpha and Beta have been frequently found to show deviations in connectivity of subjects with dementia (Tijms et al., 2013). Thus, the PLI is computed for each trial and for each signal pair after being band-passed in Alpha (8–13 Hz) and lower Beta (13–20 Hz), using an order 70 FIR filter. Note, only lower Beta could be compared across datasets due to the previously mentioned different low-pass filters implemented. These connectivity computations are then averaged over trials for each task and for each subject to remove inter-trial variability and so better bring out the specific task function. The resulting averages constitute adjacency matrices of weighted networks, one for each subject-task-frequency band triple.

Before studying the network hierarchies, the weighted PLI connectivity networks are binarised using the Cluster-Span Threshold (CST). This threshold is based on the clustering coefficient, fixing the network at the balance point of integrative and segregative properties (Smith et al., 2015). It coincides with where hierarchical information is dense, providing a sensitive and powerful binarisation of EEG PLI connectivity (Smith et al., 2017a). The hierarchy of a network is defined based on the node degrees, i.e., number of edges adjacent to each node. Nodes with more adjacent edges are higher in the node hierarchy, being more central to the network topology.

**Figure 2:**
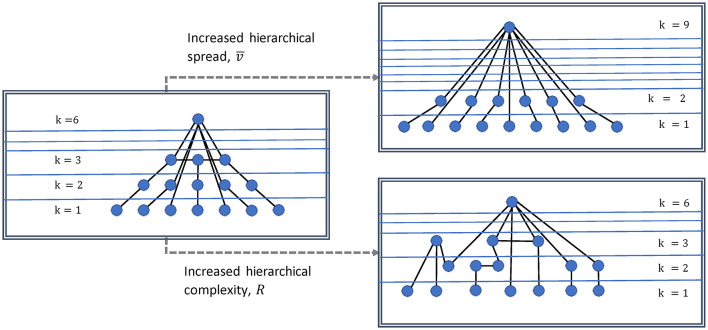
Illustration of degree variance and hierarchical complexity of a network. Increased degree variance indicates a more hub dominated network, while increased hierarchical complexity indicates a greater diversity in connectivity patterns.

We study two indices of network hierarchies described in Smith and Escudero (2017). An illustration of what these measure in a network is shown in Figure 2. The degree variance, *V*, measures the spread of the hierarchy and thus is indicative of the large range of the general strength of network nodes (Smith and Escudero, 2020) and is an important indicator of the dominance of hub nodes. Here we use the recently proposed normalized version (Mones et al., 2012):


(1) $$\begin{array}{r} \bar{v}\left(G\right)=\;\frac{n-1}{nm\left(1-d\right)}var\left(k\right) \end{array}$$

Where *k* is the variable denoting the degrees of the network, *n* is the number of nodes, *m* the number of links, and *d* the link density.

Physiologically, this measure could then inform about the expansion and/or strengthening of network structures and hierarchies. Complexity on the other hand, arises from the structure of the interactions between units (i.e., modules, nodes, or networks themselves) (Smith et al., 2019). The hierarchical complexity, *R*, is based on the diversity of connectivity patterns throughout the degree hierarchy. It is measured by the variability of neighborhood degree sequences for nodes of identical centrality (BioSemi Headcaps, 2017; Smith and Escudero, 2017). Thus, hierarchical complexity (*R*) could be understood physiologically as the level of network operational organization.

### Statistical Tests

Differences of network index values for binding and shape are computed. These differences are contrasted between patients and controls using Wilcoxon rank sum tests with statistical significance noted at the standard α = *0.05* level. The false discovery rate procedure is implemented over reported *p*-values with *q* = *0.05*. Effect sizes using Cohen's *d* are reported for significant differences. Wilcoxon signed rank tests were also implemented between datasets for the healthy controls and the MCI subjects for both shape and binding tests in order to assess whether any task activity could be discerned to be different due to aging.

## Results

### Hierarchical Characteristics of VSTMB in MCI

**Figure 3:**
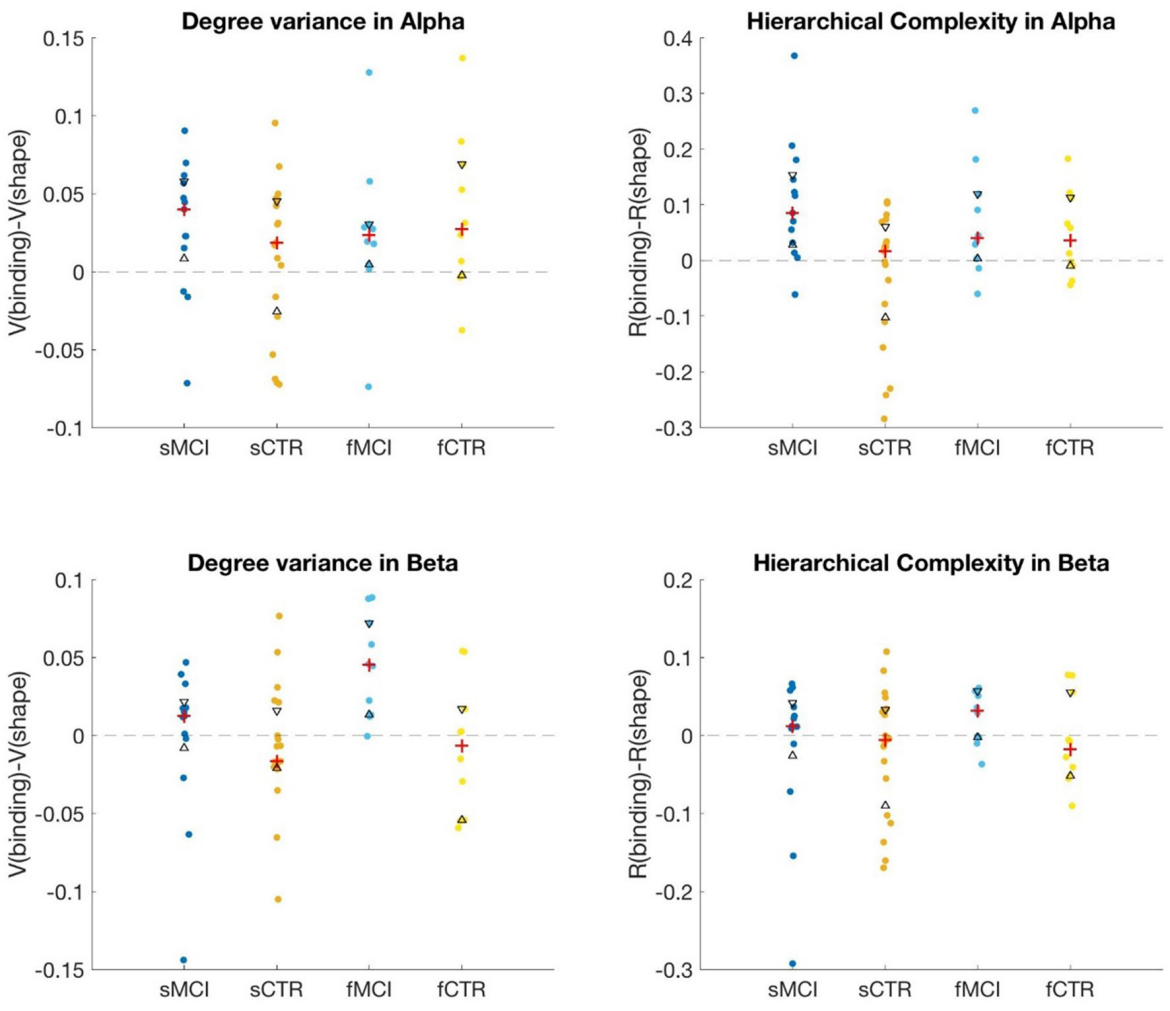
Plots showing differences between binding and shape task PLI network hierarchies in Alpha and Beta1. Red crosses indicate median values while black triangles indicate 25th and 75th percentiles. MCI and CTR indicate mild cognitive impairment and controls, respectively, while the preceding “s” and “f” indicate the sporadic and familial data.

We computed *V* and *R* for all subjects in both tasks in Alpha and lower Beta (Beta1) in both datasets. Differences were computed between binding and shape values for patients and controls separately. These values were then compared between patients and controls using Wilcoxon rank sum tests. Table 2 shows the results for each data set separately and also when healthy controls and MCI subjects in datasets are combined. A significant difference is noted in *V* in Beta1 for the familial MCI data. In 9 of the 10 patients, the degree variance of the binding condition is larger than in the shape condition, Figure 3, bottom left. Indeed, the difference in binding and shape conditions is generally greater than the difference found in healthy controls with an effect size of 1.1627. Although the trend appeared similar in the sporadic case, this was not found to be statistically significant. However, a significant difference between sporadic MCI and control was noted in *R* in Alpha with an effect size of 1.0124, which was not replicated in familial MCI and control. In 12 of the 13 patients, the hierarchical complexity of the binding condition was larger than in the shape condition, Figure 3, top right. The controls are roughly balanced between higher shape and higher binding values but larger values tended toward higher shape.

**Table 2:** Results for hierarchical characteristics of PLI networks in MCI vs. healthy control calculated from values obtained from the binding task minus that of the shape task.

		**Degree variance**		**Hierarchical complexity**	
**Test**	**Band**	***p*** **-value**	**Cohen's d**	***p*** **-value**	**Cohen's *d***
Familial MCI	Alpha	0.7337	−0.2390	0.7337	0.2739
	Beta1	0.0140^*^	1.1627	0.1859	0.6299
Sporadic MCI	Alpha	0.2827	0.3936	0.0051^*^	1.0124
	Beta1	0.2658	0.1073	0.5142	0.0492
Joint MCI	Alpha	0.6451	0.1597	0.0089^*^	0.7939
	Beta1	0.0094^*^	0.5599	0.1507	0.2266

The p-values are for Wilcoxon rank sum tests. Values with an asterisk (^*^) pass the false detection rate procedure and are deemed statistically significant.

Both the greater degree variance in MCI in Beta1 and greater hierarchical complexity in MCI in Alpha held as effects when combining the datasets. The *p-*values were roughly of the same magnitudes as in the individual dataset differences found, although the effect sizes were comparatively reduced.

### Effects Due to Aging

The network indices *V* and *R* are based on variances of degrees which can be expected to be higher for larger network sizes. Thus, to assess healthy aging of VSTM binding we downsampled the sporadic 128-channel dataset to the same size (sixty) as the familial dataset. These datasets use different layout systems for electrodes, but approximate mapping between these layouts is known (Parra et al., 2014). Following this, we reprocessed the sporadic dataset according to the 60-channel format and proceeded with comparisons. For *V* and *R*, task contrast values for each participant were attained by taking the difference of binding and shape only values. Wilcoxon rank sum tests were then conducted for older versus middle-aged adults. The results are shown in Table 3. As hypothesized, we report no significant differences in hierarchical characteristics of phase-based functional connectivity due to aging.

**Table 3:** The *p*-values for Wilcoxon rank sum tests for hierarchical characteristics of PLI networks in healthy aging and familial vs. sporadic Alzheimer's disease.

**Test**	**Band (sporadic/familial)**	**Effect**	**V (shape/binding)**	**R (shape/binding)**
Elderly vs. Adult	Alpha	Healthy aging	0.6300/0.1484	0.4490/0.6300
	Beta	Healthy aging	0.9087/0.6629	0.9451/0.5977
	Alpha	Familial vs. sporadic	0.8768/0.5558	0.7330/0.6418
	Beta	Familial vs. sporadic	0.9259/0.1629	0.3364/0.3364

*Left and right values are for shape and binding separately. No statistically significant differences were found*.

## Discussion

Research into the electrophysiological correlates of VSTM binding in healthy young and older adults and in patients at high risk of AD has revealed neurocognitive properties of this memory function that have helped explain behavioral observations drawn from these samples. Relying on novel brain network methods applied to EEG data, Smith et al. (2017b) showed that processing differences between feature bindings and shape only in healthy young individuals is driven by the effects of occipital (100–140 ms) and frontal (140–180 ms) modules over the left hemisphere. This evidence is consistent with the earlier report by Pietto et al. (2016) who, using ERP, also found that impaired function of the fronto-parieto-occipital sites accounted for binding deficits in both familial and sporadic cases of MCI due to AD. These recent studies expanded the evidence provided by previous fMRI studies (Huggins et al., 2021) which had reported a posterior parietal hub responsible for feature binding in VSTM. The advantage of the high temporal resolution of the EEG may have unveiled a wider network which functions under temporal dynamics beyond the fMRI scope. In fact, Parra et al. (2017) recently showed that focusing on such network dynamics drawn from EEG data, familial cases of MCI can be classified with accuracy levels of 90%, similar to the classification power we found here for both samples of MCI. Based on this literature, investigation of whether changes in topological hierarchies could be an additional mechanism driving the well-known VSTM binding deficits found in AD along its continuum is warranted. This is particularly relevant if we consider that it has been by means of the EEG, and not by fMRI, that an extended network subserving this function has been identified. This work complements recent classification based studies of EEG signals for AD and MCI with a greater focus on the classification of AD and MCI using machine learning methods (Yu et al., 2019; Núñez et al., 2020; Huggins et al., 2021; Miltiadous et al., 2021; Tzimourta et al., 2021).

### Hierarchical Topology in Familial and Sporadic Cases of MCI

Our results indicate that cases of MCI due to familial AD present with greater hierarchical spread (*V*) seemingly accounting for VSTM binding deficits. These results fit well the current understanding of network reorganization in carriers of AD mutation (e.g., E280A-PSEN1) prior to the dementia onset. For example, using a measure of information sharing (i.e., symbolic mutual information), Parra et al. (2017) recently reported that increased brain connectivity characterizes cases in the early stages of familial MCI while decreased connectivity was a feature of more advanced stages. Patients in the early stages not only showed increased connectivity but over-recruitment, and such changes correctly characterized 90% of the sample. The literature reporting increased connectivity and over-recruitment of task-related networks and DMN as an early feature of AD is growing rapidly (Parra et al., 2013; Gardini et al., 2015; Quiroz et al., 2015; Serra et al., 2016). This functional reorganization appears to be an early manifestation of brains undergoing neurodegeneration. This is particularly relevant in this sample of middle-age mutation carriers as they do not present with the comorbidities and risk factors that are normally associated to age. Hence, this evidence more genuinely indicates the presence of AD pathology and its neurobiological consequences.

Interestingly, AD-related network changes at older ages are characterized by greater hierarchical complexity (*R*). Hierarchical complexity is a new paradigm for brain networks which has previously been explored in EEG signals and structural MRI from healthy participants (Smith et al., 2017a, 2019) as well as on networks across broad scientific domains (Smith, 2019). It has also shown clinical relevance in structural MRI with implications for neonatal development (Blesa et al., 2021; Valdes Hernandez et al., 2021). This is the first study to apply this approach to AD. We found that such hierarchical changes were apparent in a lower frequency band (Alpha). We interpret this as the additive effects of age and AD related network changes. These findings can have some implications. Firstly, they suggest that hierarchical complexity may be an index of compensatory network coupling whereby networks operating in a particular frequency regime that becomes less efficient with aging might be compensated by networks operating at different (i.e., slower) frequencies. It is well-known that such compensation occurs at a neuroanatomical level (Heuninckx et al., 2008; Ho et al., 2012; Song et al., 2014). Based on this earlier evidence and the results presented here we feel compelled to suggest that the greater hierarchical complexity observed at a slower frequency band in age-related MCI may be informing on cross-frequency compensatory coupling. Networks operating at slower frequency bands, which remain functional in old age, may inherit the functions of decaying faster networks. Second, this evidence adds to the Scaffolding Theory of Aging and Cognition (Reuter-Lorenz and Park, 2014) as it suggests a different level of functional reorganization (see Sala-Llonch et al., 2015) characterized by cross-frequency network compensation. Finally, future research will have to investigate the extent to which these compensatory changes revealed via increased network complexity are reflecting adaptive mechanisms to cope with the effects of age, the influence of cognitive reserves, or underlying subthreshold pathology.

### Can Hierarchical Topology Help Disentangle Age and AD Related Network Changes?

Early and genetically driven AD pathology operating in younger brains alters the topology of a task-related network supporting VSTM binding by increasing its hierarchical spread (strength and recruitment) while in older brains experiencing MCI with unknown genetic factors, it hampers their hierarchical complexity. We can speculate that the latter indicates the additive effect of aging on AD pathology. Homeostatically, younger brains are better equipped to cope with pathology and to reorganize functional networks. AD impacting on older brains may encountered a less favorable biological scenario. Such a scenario has already witnessed compensatory changes (Deary et al., 2009; Reuter-Lorenz and Park, 2014; van Geldorp et al., 2014; Bastin, 2017) which are normally characterized by a loss of functional and structural network efficiency and organization. Interestingly, we found that such changes did account for VSTM binding deficits in MCI in old age, but not for a decline of VSTM binding functions due to age *per se* (i.e., we reported no significant differences in hierarchical characteristics of phase-based functional connectivity due to aging). ERP data involving patients at risk of AD due to different disease variants and with very different ages indicated that neither of these factors modified the effect that AD exerted on VSTMB, thus suggesting that its decline can be more reliably linked to the AD pathology (Pietto et al., 2016). Nevertheless, it remains of a paramount importance to develop and refine methodologies that can tease apart the contribution of AD and that of normal aging.

This is the first report of network related activity associated to the well-known insensitivity of VSTM binding (i.e., in its conjunctive form) to normal aging (Grady, 2008; Deary et al., 2009; Logie and Maylor, 2009; Reuter-Lorenz and Park, 2014; Wakefield et al., 2014; Sala-Llonch et al., 2015; Spaan, 2016). It is also the first to study network heterogeneity and hierarchical complexity of EEG networks in a clinical setting. The evidence presented here indicates that the behavioral specificity of VSTM to AD relative to normal aging also holds at the biological level when age-related compensatory changes in brain activity are considered. We can therefore hypothesize that the analysis of hierarchical topology of EEG connectivity during VSTM binding performance can be considered a potential diagnostic biomarker for AD. Nonetheless, more studies particularly with larger sample sizes are necessary to confirm these findings.

## Conclusion

We studied the functional connectivity computed from EEG during the VSTMB task in MCI stage familial AD patients and elderly MCI patients at high risk of sporadic AD as well as respective populations of age- and education-matched healthy controls. While no network differences in VSTM tasks were found due to healthy aging nor between elderly and middle-age onset MCI, clear differences in hierarchical characteristics of functional network degrees between binding and shape tasks were found in MCI but not healthy control in both datasets. It was revealed that the difference in degree variance of EEG networks in the familial AD dataset in lower Beta was significantly larger in patients with no difference found in hierarchical complexity. In conjunction, the difference of hierarchical complexity of EEG networks in the sporadic AD dataset in Alpha was significantly larger in patients with no difference found in degree variance. Combining datasets supported both increase in degree variance in lower Beta and increase in hierarchical complexity in Alpha as general characteristics of AD functional connectivity in binding. The increased complexity in elderly patients in the binding task suggests cross-frequency compensatory coupling mechanisms in an attempt to overcome the pathological damage of this disease, illuminating the possibility of a double-sided compensatory effect targeted at joint age-related and pathological decline in the brain.

## Data Availability Statement

The raw data supporting the conclusions of this article will be made available by the authors, without undue reservation.

## Ethics Statement

The studies involving human participants were reviewed and approved by Ethics Committees of the University of Antioquia and INECO. The patients/participants provided their written informed consent to participate in this study.

## Author Contributions

KS conducted the data analyses, wrote the manuscript, did the literature search, and produced the figures. JS contributed to the study design and writing. JE contributed to the study design and data analyses and revised the text. AI was involved in collecting and providing the data and revised the text. MP designed the study, interpreted the findings, and contributed to the writing. All authors contributed to the article and approved the submitted version.

## Funding

KS reports grants and personal fees from Engineering and Physical Sciences Research Council, and grants from Medical Research Council during the conduct of the study; AI reports grants from Takeda CW2680521; CONICET; FONCYT-PICT (2017-1818, 2017-1820); ANID/FONDECYT Regular (1210195, 1210176, 1220995); ANID/FONDAP (15150012); ANID/PIA/ANILLOS ACT210096; and the Multi-partner Consortium to expand Dementia Research in Latin America (ReDLat), funded by the National Institutes of Aging of the National Institutes of Health under award number R01AG057234, an Alzheimer's Association Grant (SG-20-725707-ReDLat), the Rainwater Foundation, and the Global Brain Health Institute. MP work was supported by Alzheimer's Society grants AS-R42303 and AS-SF-14-008.

## Author Disclaimer

The content is solely the responsibility of the authors and does not represent the official views of these institutions.

## Conflict of Interest

The authors declare that the research was conducted in the absence of any commercial or financial relationships that could be construed as a potential conflict of interest.

## Publisher's Note

All claims expressed in this article are solely those of the authors and do not necessarily represent those of their affiliated organizations, or those of the publisher, the editors and the reviewers. Any product that may be evaluated in this article, or claim that may be made by its manufacturer, is not guaranteed or endorsed by the publisher.
